# The Frailty of the Invincible

**DOI:** 10.37825/2239-9747.1000

**Published:** 2020-10-01

**Authors:** M Illario, V Zavagli, L Noronha Ferreira, M Sambati, A Teixeira, F Lanata, S Pais, J Farrell, D Tramontano

**Affiliations:** 1 Health Innovation Unit, General Directorate for Health, Campania Region, and Federico II Department of Public Health, Naples, Italy; 2 Psycho-oncology Unit, ANT Italia Foundation, Bologna, Italy; 3 Centre for Health Studies and Research-CEISUC, University of Coimbra, Coimbra, Portugal; 4 Gruppo Cassa depositi e prestiti, Rome, Italy; 5 Research Unit for Sport and Physical Activity, Faculty of Sport Science and Physical Education, University of Coimbra, Portugal. ateixeira@fcdef.uc.pt; 6 Wind & Sun Foundation, Genoa, Italy; 7 Comprehensive Health Research Centre (CHRC) and Algarve Biomedical Center (ABC)-University of Algarve, Faro, Portugal; 8 EIP on AHA RSCN Bruxelles, Belgium, and LANUA International Healthcare Consultancy, Northern Ireland; 9 Dpt of Molecular Medicine and Medical Biotechology, School of Medicine, University of Naples Federico II Naples, Italy; 10 Italy and GENS Onlus Foundation Naples, Italy

**Keywords:** inequalities, well-being, reconstruction

## Abstract

The COVID-19 pandemic has unveiled the frailty of our societies from too many points of view to look away. We need to understand why we were all caught unprepared. On the one hand, we have all short memories. As we forget too quickly, we were unable to recognize key factors influencing response and preparedness to public health threats. For many years, economic evaluation pushed governments all over the world to cut resources for public health systems, with COVID-19 pandemic the question arises: do we spend too much or too little on health care? What is the right amount to spend on health? Moreover, in many countries, the privatisation, or semi-privatisation, of healthcare may give rise to inequitable access to health care for everyone. Although COVID-19 is very “democratic”, its consequences aren’t. According to OECD, income inequality in OECD countries is at its highest level for the past half century. Three main causes have been recognized, technological revolution, globalization, and “financialisation”.

In this scenario, lockdown measures adopted to save lives are showing dramatic economic consequences. To address post COVID-19 reconstruction we need to go beyond GDP. As an economic measure this has many shortcomings in describing the real well-being of a country, and since what we measure affects what we do, new paradigms will have to guide the post COVID-19 reconstruction strategies, as the fate of countries and their citizens is at stake.

## INTRODUCTION

Thucydides (460–395 BC)

### *History of the Peloponnesian War*, (431–421 BC) Plague of Athens II.vii.3–54

«[…] The doctors were unable to cope, since they were treating the disease for the first time and in ignorance: indeed, the more they came into contact with sufferers, the more liable they were to lose their own lives. No other device of men was any help. Moreover, supplication at sanctuaries, resort to divination, and the like were all unavailing. In the end, people were overwhelmed by the disaster and abandoned efforts against it.[…]»

### Frailty at the times of COVID-19: an overview

Frailty is a current reality. Unprepared to face this “black swan” COVID-19 has highlighted our frailty as individuals and as society [1,2]. The speed and spread of COVID-19 has engaged us (Governments, Health and Care Providers, Scientists, Health Professionals, and society as a whole) in a race against the clock to limit the damage, to save lives! Science is working hard to “understand” this new threat in order to develop a new vaccine and antibody tests, but it takes time [3–7]; governments are trying to stop the spread of epidemic, but it will take time in order to see if the containing measures are effective; health systems all over the world are continually under pressure and in some cases almost at the point of collapse, but to build new intensive care units takes time. Meanwhile society is scared, confused, and looking to politicians, scientists and medical professionals to provide leadership and a managed response. We believed we were invincible, untouchable, immortal. In the wake of COVID-19, we now understand what it is to feel frail and have all our certainties jeopardized or taken away from us. Our mistake from the beginning, repeated over and over again, was to reject the unthinkable. We knew from scientific evidence that the world would have faced another pandemic, as we were alerted in recent years by SARS, MERS, H1N1[8], but what did we learn from these and what did we do with this learning in preparing for the future? In a heartbeat the unthinkable broke into our lives. Just like this insidious virus, the unthinkable already pervades every fold of our daily lives. We never expected to need a justification for throwing away garbage. We would not have expected to regulate our days around the spread of the virus or the daily reports on the increasing number of deaths. We could not imagine someone dying without the people they love beside them; that those left behind would grieve by themselves without other family members and friends for support; or that funerals would be silent and deserted. What we considered an achievement, or took for granted, like enjoying the experience of travelling, our lifestyle and interaction with other people, going to a movie, or going out for dinner with family and friends, suddenly presented a danger. Moreover, ”we thought we could be healthy in an unhealthy world” (Pope Francis, March 27 2020).

COVID-19 took us back in time, and in a world of technological and intangible values, we returned to reasoning about the movements of human beings, and the power of contagion.

To face COVID-19 in 2020 we are using the protocols of our Medieval ancestors, their heritage engraved in our collective memory. Our ancestors had no medical cures, they just tried to react by defending themselves. Once they understood the disease was highly contagious, they adopted defense measures: to let people know immediately what was happening; isolate the sick and vulnerable; close the areas of infection; not hold funerals; etc. The concept of “Quarantine” was developed in Venice during the first great plague of 1348, where it was used to describe the period of 40 days a ship suspected of carrying a contagious disease was held in offshore isolation [9] Almost 700 years later the term is being repeated in a digital era.

The truth and reality may be difficult to accept, but COVID-19 has caught us totally unprepared. Being unprepared, we were slow in reacting; and this delay along with the lack of plans, actions, and response measures could be viewed as contributing to the global spread of the infection. The result of global spread can be viewed as a sanitarian, humanitarian and socio-economic catastrophe, the extent of which is difficult even to conceive.

It seems incredible, but countries that have been talking about security for years – building walls, raising barbed wire barriers, building one “false flag” operation after another; used these tactics to create internal fears and anxieties and *create enemies*; to shred social fabric, and undermine social cohesion and solidarity. All those countries have discovered that viruses are “democratic” and they do not care about any sort of differences between people or between countries; a virus does not recognize or indeed care about boundaries, walls, barbed wires; no one is untouchable; no one can win this kind of war alone. There are no positive aspects of COVID19, an evil that has already taken the lives of thousands of people, but there are lessons to be learnt.

### We need to understand why

The physician and philosopher Wilhelm Wundt Heterogonie der Zwecke (1832 – 1920) described precisely what is happening today as we face up to COVID-19; our global *unpreparedness* «unintended consequences of intentional actions ».

Analyzing the causes is not a sterile, pointless exercise nor the obsessive search for the guilty, or a scapegoat to blame. In one way or another we are all guilty perhaps through our own negligence, a lack of interest in what is happening around us, miscalculation, or ignorance, to list just a few traits.

As a society we need to know and to take responsibility for the causes. By recognizing and addressing them we can lay the foundation for our future. Nothing will be like before, as scary as this may sound, Covid-19 may be a watershed. Whether we will have a future and what “the after” could look like, depends only on the choices we will make now. The old “Normal” will be replaced by a new “Normal”, something that will be shaped by the actions we take now. Normal will no longer be something we accept or conform to because others tell us; it will be a state that continues to evolve and develop as we apply learning and experience to the type of society we want to create. One thing is crystal clear: there will be no winners and losers, either we will all win or we will all loose. At stake is our fate.

We cannot deny that mistakes have been made and acknowledging that would be the starting point. Reviewing our actions, or inactions, and learning from our mistakes, and successes, is a key step to avoiding repeating the mistakes of the past. Unknown Corona viruses are present in wild animals, together with unknown bacteria and parasites. Deforestation, intensive breeding and climate change are considered factors that can contribute to increasing the possibility of spillover to humans. Thus, we cannot realistically imagine that COVID-19 is an exceptional event, but it could be a hard lesson to learn.

### Short memory: we forget too quickly

The success against infectious diseases achieved with vaccination, and the eradication of smallpox, perhaps gave us an unfounded confidence that infectious disease would no longer be a problem for human beings [10]. This may have been more evident in the past 20–25 years because of the explosive cocktail of medicine advances, low mortality at birth, population ageing, increases in non-communicable chronic diseases like cardiovascular and degenerative diseases, diabetes, obesity and cancer [11]. Setting aside the individual impact from the latter, they have been leading to additional pressures on our health and care systems and prompted a shift in research funding away from infectious diseases, despite recurrent public health emergencies occurring in sub-Saharan Africa and in other low-income countries worldwide. The increased of incidence of no infectious diseases and their impact on citizens and society also led to increase in investment in research funding, and to changes to healthcare policies and service delivery models. Similarly, health prevention measures in medium and high-income countries were focused to reduce Non Communicable Disease. We went through the epidemiological transition from communicable diseases to non-communicable disease [12], Not even the onset of SARS (2002–2003 Infected 80,989 deaths 774), MERS(2012–2019 Infected 2,494 deaths 858) and Ebola (2013–2016, Infected 28.646 deaths 11.323) epidemics [13–15]apparently shook our certainty that infectious diseases were behind us, in spite of the warnings from the WHO and from the scientific communities. WHO has outlined very carefully how to proceed when an infectious outbreak occurs, providing procedures, guidelines and tools for preparedness responses that have not been adequately taken into account. [16–18]

Special mention needs to be made of SARS, as it was a true ‘globalization epidemic’, which used the speed of travel and mobility of the population as its diffusion system. When the disease was defeated it was thanks to the combination, in some paradoxical way, of the oldest and most modern health methods-biomedical and IT technologies, in particular “data sharing”. Consequently, SARS’ short history dramatically demonstrates the global risk represented by the emergence of new diseases, but at the same time showed clearly the essential role played by the availability, collection, interpretation and dissemination of ‘open data’.

Some countries like Canada learned the lesson, and in 2004 the national report of the National Advisory Committee on SARS and Public Health in Canada evaluated the lessons learned from public health interventions to contain severe acute respiratory syndrome and offered advice for future infectious disease control and prevention. The Committee set out a plan for a comprehensive renewal of both the public health system in general and the nation’s capacity to detect, prevent, understand, and manage outbreaks of significant infectious diseases [19]. In spite of several early mistakes, China successfully reacted against the epidemic. In hindsight the reason is clear: China had successfully eradicated SARS and the Chinese had learned the lesson well: when central power became aware of the situation, it acted with the readiness of those who had plans ready. The same can be said for Singapore, Hong Kong, Taiwan, and South Korea which “benefited” from the SARS outbreak in 2002–2003, by upgrading their institutional readiness.

It is of note that although SARS, MERS and Ebola, crossed land borders from their places of origin, the infection and toll was confined mainly to Asia and Africa. Viruses and germs do not respect borders, social and geographic, which means our resources, including scientific and technological knowledge, are stuck at the borders between rich and poor countries. This raises a fundamental question about the inequitable distribution of technological, financial and human resources across the world [20].

Neglecting, overlooking or just forgetting the warnings about emerging diseases, can be no excuse when setting the priorities for research, medical and public health studies; or developing the content for undergraduate and post graduate medical or scientific programs. Doing so, as evidenced by COVID-19, implies a gap of knowledge, experience, and competence in understanding emergent diseases for which we were caught unprepared.

We also need to understand the implications from reductions to research funding which deny scientists the opportunity to investigate emerging infectious diseases and to develop potential treatments. However, we cannot underestimate the ideological positions, emerging from anti-science lobbies, e.g.. no-vax and animal right activists, have had on influencing political decisions to impose limitations on experimental animal studies, and the ban of fetal tissue. This has contributed to actually blocking and delaying the study of potential coronavirus therapies and vaccines) [3–7, 21].

### Key factors influencing response and preparedness to public health threats

Reflecting on this it is therefore not surprising that decision makers in government were widely unprepared on how and when to address COVID-19, and thus contributed to dramatic delays in the response to the virus outbreak. Consequently, when everything is considered, the difficulties experienced by healthcare systems in dealing with Covid-19 appear understandable, at least in part. Financial reductions in healthcare budgets and personnel can also explain the other, main, part of the problem. For many the delivery of publicly funded healthcare may be considered an unbearable financial burden, leading to public health care systems experiencing significant reductions, in real terms, in the allocation of public resources[22]

### Do we spend too much or too little on health care? What is the right amount to spend in health?

Different answers are given by different nations who do not always understand the economic loss from an illness is always greater than the total cost of treating it. Unfortunately, the idea of the primacy of the economy for which, in recent years, closing a hospital to save money, or not investing in health to sufficiently reflect demographic changes has always been economically justified; however, today it could be seen as a major contributor to creating frailty in our society. The Covid-19 pandemic toll stands to remind all of us that healthcare is an investment for a more productive society, for example it is central to creating and maintaining a healthy workforce; it acts as an economic generator because of the direct and indirect jobs it creates, and the opportunities it can provide for industry and SMEs in providing goods and services. It also provides research opportunities leading to the development of new solutions and technologies to address health diseases and support new ways of working. We need to change the view of healthcare and not see it simply as an economic burden on society, but rather as an engine for economic growth and health improvement. This is not to say that we divert attention from its real purpose, looking after the health and well-being needs of the population - this should always be the primary focus - but we should acknowledge that, by investing in health and care, we can create both a healthier society and economic opportunities.

“Individually and collectively, Our Health is Our Wealth” [23]. Preventing diseases and promoting health is even more valuable to individuals and to society as a whole. Today, all at once we find that in responding to COVID-19 financial rules and the economy, which have been at the forefront of political decisions, have been put in the background as Governments attempt to regain control over nature. Decisions are being made now that can have long term financial implications for countries, but these actions, and not the cost of doing them, are fundamental to saving lives. “Economics first” doesn’t work, neglecting and/or overlooking population health will always be a cost on society. In fact, health and economics cannot be seen as having opposite interests. In fact, health and economics have been holding hands for the last two decades, since the great development that health economics has faced. Economics can be seen as “a science which studies human behaviour as a relationship between ends and scarce means which have alternative uses” [24]. As available resources are never enough to address all societal’ needs, these concepts of scarcity and choice are very important when planning health policies and provision of healthcare. Therefore, choices have to be made and they may involve very difficult decisions. This is why since the 1970s a set of analytical methods of economic evaluation have been developed to inform decision-makers on how the scarce health resources can be allocated in the best way to maximize the health gain. Economic evaluation compares alternative courses of action in terms of both their cost and consequences, having in mind that if the resources are used in alternative A, they will not be used in alternative B. This does not mean that a monetary or a strict economic vision is used, since the preferred type of economic analysis used in healthcare is cost-utility analysis, where cost and consequences are compared, but consequences have to include not only the benefits of an intervention in terms of measurement units (cases detected, number of lives saved, cases prevented, etc.), but also its effect on survival measured in terms of life-years, and on quality of life. Over the last two decades, economic analysis models have been used in almost all developed and developing countries to inform the development of policies by decision-makers. Health authorities around the world have, since the early 1990s, started to demand that economic evaluation studies are carried out for the purpose of calculating the return of investment from medicines and other health technologies, in determining healthcare budgets and maximizing health gains. Therefore, health is a sector where decisions are made, in most countries, within perfectly defined criteria, which are clear and take into account the quality of life of the population and their well-being. However, many times criteria that have been so well defined and applied are not used by decision makers at the macro level to help in allocating investment decisions across economy sectors. In fact, this exceptional situation society is now facing caused by the Covid-19 outbreak has highlighted the need for carefully evaluating allocation decisions at a macro level, in order to use the scarce resources in the most efficient and effective way. Decisions to invest in different areas of the economy and society should therefore be based on more rigorous criteria, based not only on cost, but on the true benefit for society. In fact, what should be done are real economic evaluations on investments across sectors, given the cause and effect of such decisions. For example, where there are competing investment decisions to be made, policy makers should adopt a holistic approach measuring the impact of the investment not just in economic terms but also against other societal indicators such as health, climate, etc where the benefits may not be realised for a number of years and where the impact on the population is over a longer timeframe. Such benefits cannot be presented simply as financial profits or income generation but should be seen as long-term savings to the health and care system and developing a healthier economically active population which can support economic growth. We must look at the economy and society’s needs as whole, and we need to decide what is the most efficient and effective way of spending our resources so they have the greatest effect on society. The reduction in resources to deliver safe and effective care has led to the weakening of health and care structures, depleting and scaling back services, and in reductions to clinical, nursing, and health and care professional staff. COVID-19 has helped to highlight the impact from inadequately funded health and care services. Although only a fraction of COVID-19 cases (around 8%) require hospitalization and Intensive Care, we have seen evidence across all countries dealing with the pandemic that the increased daily admissions to hospitals has only added to the already over-stretched capacity. In spite of the efforts to equip new ICUs, to build new COVID-19 emergency hospitals and field hospitals, it is a reality that exceeding hospital capacity and creating additional pressures on already over-stretched clinical and nursing teams will have tragic consequences. Not just for patients but also for those looking after them.

### The prominent role of business in public life

Since the 1980s, governments have for the most part let business steer and create wealth, intervening only for the purpose of providing financial aid, supporting the development and opening of new markets, or fixing problems when they arose. In this process, critical institutions providing public services and public goods more widely are left weakened. The prominent role of business in public life has also led to a loss of confidence in what the government can achieve alone – leading in turn to the many problematic public-private partnerships, which prioritise the interests of business over the public good. Privatization of health care is not homogeneous everywhere, and in some countries it may lead to those on low income being without any health care protection. In simple words we are lacking the only protection we need and which really matters-social protection, an essential element of social cohesion.

### The rise of inequalities

According to OECD, [25] income inequality in OECD countries is at its highest level for the past half century. The average income of the richest 10% of the population is about nine times that of the poorest 10% across the OECD, up from seven times 25 years ago. Moreover, in between the richest and the poorest is the “middle class”. In many OECD countries, middle incomes have grown less than the average and in some they have not grown at all. To understand how, why and the significance of the middle-class decline, it is necessary to go back to the end of WWII. In the decades following World War II, the world experienced a phase of unusually strong economic growth, which came to be known as the Golden Age of Capitalism. The global growth rate averaged almost five percent per annum. During this period the state also complemented markets in a way that had widespread benefits throughout the economy. Europe, which was reconstructing itself after the devastating effects of WWII, witnessed fast-paced economic growth aided by the U.S. under the Marshall Plan. A system of progressive taxation across these countries also played a vital role in ensuring equitable distribution of wealth during this period. However, growth slowed with the 1973–75 recession sparked by the oil crisis, the world economies began looking for avenues to kick-start the growth process again and turned to globalization. The United States, and European countries, initiated liberalization at home and deeper economic integration with one other. Over time, the developing economies also opened up their markets. Economics is not an experimental science in the strict sense as it has been known for over 200 years. It is not possible to experiment “in vitro” just to see how the economy would react if certain parameters were changed instead of others, and the alternative would be to try to do it in reality, at the risk of a little social butchery. The benefits of globalization and liberalization were not as expansive as they were in the post-War era. Globalization did extricate the highest number of people out of poverty in human history, but the developing countries were the biggest beneficiaries. The Western world, in contrast, hardly benefitted from the process of globalization. In the developed countries, middle class income stagnated. While worker productivity continued to grow after 1973, the growth in their compensation stagnated. Two factors played a key role in wage growth stagnation. The advent of technology led to widening the gap between productivity of the economy and the compensation paid to workers. The technological revolution since the early 1980s pushed economies towards automation and greater use of ICT benefitted those with higher skills disproportionately. Moreover, many low-skilled jobs have vanished over time due to technological interventions that have not been paralleled by adequate training approaches for the workforce.

Moreover, the technological advances in both logistics and telecommunications, together with the liberalization of trading and of the financial movements and activities, has resulted in an increase of the average transaction speed, with the result that profits from trade and financial transactions are accrued at a much quicker rate, improving the profit ratio of purely trading and financial transactions, at the detriment of productive industrial activities. This has been compounded by the financial liberalizations, exacerbated by the very strong profit competition in the financial field, The “financialisation” level (percentage of GDP due to financial transactions and activities, i.e. deriving from activities not directly correlated to actual production) is higher than average in some of the world’s national economies, at the same time, those are the economies showing the highest level (among developed countries) of income inequalities, when compared to same level economies, which maintained a higher level of industrial activity, such as Germany.

Despite inequalities rising across the developed world since early 1980s, the final straw that broke the camel’s back for the segment of population that was left behind was the financial crisis of 2008. It was a crisis created by the rich, yet they were bailed out and suffered the least due to it. Those who had not benefitted from the boom bore the consequences disproportionally in terms of joblessness and lack of economic opportunities. To address the crisis, central banks across the developed world kept the interest rate near zero and bought trillions of dollars in bonds to encourage spending. But instead of creating employment and fueling wage growth, much of that money ended up driving the value of financial assets upwards. Since the rich own such assets in higher proportion, inequality has only accentuated since the crisis and furthered the disenchantment of the people. Faced with impoverishment, middle classes have reduced their ability to save, and in some cases have fallen into debt. Moreover, worst of all, middle-class saw the dream of a better life for them and for their children vanishing, for them the social elevator got stuck. Instead of upwards social mobility and growing prosperity, the middle classes became more worried about slipping downwards and this lack of optimism for the future is echoed in other signs of social distress. The stagnation of middle-class living standards, uncertainty and fears of social decline and exclusion standing out against the background of the emergence of new forms of nationalism, isolationism, populism and protectionism. Middle classes, the “bedrock of economies and of democracy”, traditionally having a moderate feeling of being “left behind” are increasingly likely to support “anti-establishment” movements. This can have dramatic consequences on social cohesion and social capital as it undermines trust at both the individual and collective level. Social cohesion is the “glue” of a society, consolidating plurality of citizenship by reducing inequality and socioeconomic disparities and fractures in the society. It reflects people’s needs for both personal development and a sense of belonging and links together individual freedom and social justice, economic efficiency and the fair sharing of resources, along with pluralism and common rules for resolving all conflicts. Social cohesion finds its foundation in an equal society which ensures economic equality and equality of opportunities [26].

Destruction of social cohesion jeopardizes democracy as there is no democracy without social cohesion. Moreover, it has been shown that increased trust has the same impact on life satisfaction as an increase by two-thirds of household income [27]. A positive relationship between well-being and overall social cohesion has also been established [28]. Finally, social cohesion fosters mental [29] as well as physical health, even moderating the effect of income equality on increased mortality. It has also been demonstrated that a disinvestment in social capital leads to the rise of mortality rates [30]. Thus, arresting the rising of inequalities has to be the priority for policy makers in the COVID-19 era.

### The present round of globalization, under a technical, economic and societal perspective: a use case scenario

The present round of globalization has deeply impacted western societies although to different extents, across different regions and industries. An example of those differences could be the present situations of the automotive and tourism industries in the USA, and the air travel and steel industries in the EU. In that, while the automotive and steel industries have suffered from competition coming from developing world countries, both the air travel and tourism industries have instead received a boost from globalization [31]. In addition, globalization in general has contributed to polarizing societies, and endangering social cohesion.

To get a deeper insight on the “how and why” of globalization impact, we can consider as a possible case study, the Port of Genoa.

The Port of Genoa, and its associated logistic industry, is the largest Port in Italy, and of the Mediterranean Sea, it is also a hub of logistics and other services, directly influenced by globalization, as well as being an important industrial center. The Port and logistics industry has been and still is a mainstay of Genoa’s economy, since medieval times together with its ancillary and dependent functions (i.e. insurance, shipbuilding, maritime law), and it has produced a healthy and proud middle class, coming both from the ranks of the “Port Companies” of longshoremen (i.e. the more specialised trades, among them stevedores, tallymen, gang chiefs) and from the ranks of freight forwarders, insurance companies, terminal operators, tugboat companies, Port Pilots and so on. All these “trades” represented a pillar of the city of Genoa’s economic and social fabric. They were very attentive to the “wellbeing” of their members, and shareholders, who were supported through a network of social and cultural initiatives such as mutual help funds, health funds, people’s and itinerant libraries, popular universities, amateur theatre societies, and many other offerings, often derived from much older medieval institutions.

We will discuss the technical evolutions that made possible the present level of globalization, its effects on the Port, logistical and service industries activity, and the consequent social effects.

Up to the end of the 1970’s, the typical general cargo vessel, calling at Genoa as at any other port, carried less than 10,000 Metric tons of cargo, packed in crates, barrels, boxes, bundles and cases, which had to be loaded/unloaded individually, by means of cargo nets and/or crane hooks and slings, into sorting warehouses ashore, with few exceptions. The single cargo parcels were, for each single piece, identified by the “markings”, and if “inbound” had to be sorted before being delivered ashore to the receivers. That meant that a single cargo vessel could unload/load a maximum of about 1,000 Metric tons of cargo per day, employing on board 80 to 100 longshoremen, plus 10 to 12 highly skilled auxiliary personnel such as artisans for repairing broken crates/barrels etc, crane handling, tallymen for checking quantities unloaded, forwarders and the like, for at least 10 working days. The number of skilled personnel involved when a ship docked was huge, since every single item of cargo had to be properly stowed on board, for loading, or properly extracted from the hold, to unload it – and that alone required a high degree of experience and empirical training. The costs were high but productivity was low. Longshoremen were considered a “labour aristocracy”, very well paid and independent, since they were, by law, employed only by their cooperative. The same was true for most other port professions.

Supporting businesses(shipping agents, ship forwarders, clearing agents) all had to process by hand intricate and high quantities of data, often under pressure, and a mistake could have very serious commercial and legal consequences. This meant that they too had to be highly qualified, for that time, which meant they too enjoyed a high status and pay.

With the introduction of containers in the mid-1990s, the carrying capacity of a single container ship was about 20 times the carrying capacity of one vessel of the 1970s.

One of these container vessels can load or unload, each operating day, somewhere between 50,000 to 60,000 tons of cargo, with less than half the number of people, requiring lower qualifications and skills, than those employed in the 1970’s. The same is valid for the “ancillary or support” operations, since the exchange/issuing of bills of loading, cargo manifests, invoices, custom clearance documents, onward forwarding and delivery instructions, stowage plans, ship’s documents are all heavily computerised, automating almost completely the previous (and well paid) clerical and middle management jobs. This has led to a huge productivity improvement, making it a commercially viable proposition to increase trade and, in conjunction with the decreased costs of worldwide telecommunications, also makes it possible and logical (from a purely business point of view) to widen the locations of historical industrial production centers, which previously were concentrated in the industrialized countries.

The timetable of this revolution starts from the beginning of the 1970’s, with the first instances of container ships in service, the mid 1970’s with the first communication advances e.g. Fax machines, the 1980’s with the first instances of EDI ( Electronic Document Interchange, with EDIFACT a NU standard), the 1990’s advanced telecommunications and the growth of IT leading to a reduction in costs.

Following the third industrial revolution “trades” have been hugely reduced in numbers, and their average pay has been reduced, relative to the level of the average industrial worker.

Those changes have had a social impact both at the individual level undermining “trades” self-esteem, and at a collective level as these “trades” no longer have the same recognition within their social context. Moreover, the social and cultural support network started to fall apart. Embedded in the theory of the social determinants of health is the concept of social and community networks – trusting relationships that allow people to support one another and in so doing promotes quality of life. In addition, social interactions and support systems play an important role in overall health. At the same time, a much lower number of “top managers” have hugely increased their relative social and economic importance, which in parallel to the impoverishment of the “trades” contributed to society polarization, with consequence on the entire social context and jeopardizing social cohesion. In turn, “ top managers” quite frequently expatriate, working for non-EU countries, contributing to the impoverishment of the local social capital.

### The lockdown and its economic consequence

The life-and-death imperative posed by COVID-19 forced governments, and the more skeptical, to impose restrictions on the movement of people and suspend “unnecessary” commercial activities in order to lower the rate and spread of infection. Such perceived draconian public health measures bring us inevitably to speculate about the complex relationships between health and economics. This is likely to have a stronger focus as countries begin to exit the pandemic as the IMF, and others, are advising of a significant economic recession caused by the impact of the pandemic on global economies (Governments are preventing workers from working - deepening the supply-side recession; and citizens from spending - deepening the demand-side recession); resulting in financial interventions by National Governments, and the European Union to try and stabilize their economies in the short-term. This recession, in other words, is an unintentional consequence from an unavoidable intervention to support the economy.

### Beyond GDP

Economists recognize that COVID-19 will dramatically impact the global economy and extraordinary measures will be needed as soon as possible after the pandemic to limit the economic damage and reduce a global recession.

To do so we need to rethink the basis of economic strategies. We need to expand our measure of development so that it takes into account a further indicator, society’s quality of life. As we consider the economic costs from a pandemic, making reference to COVID-19, perhaps now is the time to acknowledge the limitations of GDP, and to consider a new formula GDP, one that recognizes and takes account of the true indicators for economic and society well-being.

Gross Domestic Product (GDP) is the monetary value of final goods and services produced in a country in a given period of time and is used as the main indicator of the economic performance of a nation. Although generally adopted as a measure of a nation’s development, long time critics of GDP have argued it is a fairly narrow metric which was developed for economies were “goods” had primacy over “services”. Indeed, Simon Kuznets, who developed the modern concept of GDP for a US Congress Report in 1934, warned against its use as a measure of welfare: “The welfare of a nation can scarcely be inferred from a measurement of national income” [32] and in 1962 further stated “Distinctions must be kept in mind between quantity and quality of growth, between its costs and return, and between the short and the long term. Goals for more growth should specify more growth of what and for what”[33]. In later years Robert. F. Kennedy, in a famous conference given on March 18^th^, 1968 at the University of Kansas said: “Yet the gross national product does not allow for the health of our children, the quality of their education or the joy of their play. It does not include the beauty of our poetry or the strength of our marriages, the intelligence of our public debate or the integrity of our public officials. It measures neither our wit nor our courage, neither our wisdom nor our learning, neither our compassion nor our devotion to our country. It measures everything, except that which makes life worthwhile.” [34] GDP therefore does not measure inequalities, nor costs such as pollution and depletion of nonrenewable resources, social capital, leisure time, quality of life - in a word well-being [35,36]. After the 2008 economic crisis, the question whether monetary measurements alone, i.e Gross Domestic Product could still be considered a comprehensive measure of national prosperity exploded again among scholars and policy makers. A critical report of GDP was published by The International Commission on the Measurement of Economic Performance and Social Progress, “*Mismeasuring Our Lives: Why GDP Doesn’t Add Up”*. It concluded: GDP is not a good measure of wellbeing. What we measure affects what we do. If we measure the wrong thing, then we cannot be surprised if we continue to do the wrong thing.[37, 38] If we focus only on material wellbeing – the production of goods, rather than on social well-being - health, education, and the environment – we distort the understanding of a nation’s true wealth and growth. Measuring the ‘busyness’ of the economy and calling that progress never was and never will be the route to a lasting prosperity for all. Focusing exclusively on GDP and economic gain to measure development ignores the negative effects of economic growth on society, such as climate change, income inequality, health inequalities, etc. It’s time to acknowledge the limitations of GDP and expand our measure of development so that it takes into account a society’s quality of life. In simple words “GDP is dangerously inadequate as a measure of quality of life” [39]. What is meant by “quality of life”? Simply put, it is the standard of health, comfort, and happiness experienced by an individual or a group. Quality of life is a very important concept that needs to be taken into consideration in all sectors of the economy. In health economics it is taken into consideration within decisions on the financial return from pharmaceuticals and other health technologies; it is time for it to be also considered in other financial allocation decisions within the health sector and across other sectors of the economy.

### Informal Caregiving as an example of GDP inaccuracy

In many countries unpaid family caregivers constitute a substantial part of the total care received by chronic and terminally ill patients of all ages. Informal care makes an important contribution to societal welfare, complementing and substituting the formal care patients receive. Caregivers provide a mix of care activities, such as household work (cleaning, cooking, groceries shopping), personal care (dressing, washing, feeding) and practical support (moving outside the house, going to the physician). Many of these family caregivers also provide health-related services, assisting patients with the management of their disease and helping them to cope with emotional distress. Therefore, caregiving is burdensome and there is increasing evidence that providing informal care may lead to health problems, both in terms of morbidity and mortality.

Demand for informal care is likely to increase in the future, due to the aging of the population, and the rationing of formal care support in many countries.

Despite its contribution, informal care is often neglected in economic evaluations. Eisler highlighted how the GDP does not account for the economic activities that exist outside the realm of monetary exchange, such as “the caring economy” [40]. Other authors argued that GDP is an inadequate economic index and that it simply fails as a measure of societal welfare.

The neglecting may be related to the lack of a standardized methodology in definition, measurement, and valuation of the impact of informal care in economic evaluations. In fact in literature different available methods are discussed. The debate emphasizes the fact that time is not easy to value and similarly it is not simple to identify and evaluate the full costs and health effects of assistance for the family caregivers [41]. In this respect, some studies have highlighted that the degree of dependency and the formal care received [42] were the main variables explaining the variability of caregiving time provided.

Despite the ongoing debate regarding methodological questions [41,43] some studies have tried to make economic evaluations of informal caregiving. For instance, a study rated that the value of informal care provided by main caregivers in the U.S. in 2006 represented about 2.7% of the GDP ($13 trillion) [44]. Oliva-Moreno et al. [45]the total value of informal care was ranging from 1.73% to 4.90% of the national GDP in Spain in 2008 (monetary valuation ranged from €32,164 million to €53,299 million. In Italy, the estimated number of hours of informal care provided in 2015 was 7,954 million whose monetary evaluation amounted to €77,713 million, representing 4.73% [46] of the GDP.

Certainly, as already mentioned, more research is needed and it would probably be useful to combine different methods. There is no doubt, however, that a full evaluation of the costs and effects of providing informal care is necessary. In fact, ignoring informal care is problematic, because it may result in biased policy recommendations and decisions. On the contrary, inclusion of caregiving is crucial to promote caregivers’ social recognition and it is important to accurately inform decision makers about costs, savings and use of formal and informal healthcare resources [43]. It would also permit adequate funding for family caregivers’ support to be obtained, and this would provide an excellent return on investment. In fact, providing them with better assistance can have positive consequences on the social welfare, the health care system, and the economy in general. This is because if family caregivers maintain their own physical and emotional well-being, they can provide the best care possible to the patients.

Therefore, we should take informal care evaluation seriously, keeping in mind the words of Christopher Hoenig: “the world is truly what we make it, and how we measure it.”[47]

### COVID-19 pandemic: The collateral effects on health

Almost everywhere hospitals and more generally the healthcare systems have been choked by COVID 19; patients have been forced to delay or postpone assistance, and the treatment of people affected by other diseases, like cancer, or on a waiting list for surgery or diagnostic intervention is being delayed. This will result in a further increase in deaths, this time as a consequence of COVID-19 and not as a direct result of COVID-19. Moreover, the COVID19 pandemic will inevitably have other consequences on population mental and physical health and well-being. It is of paramount importance Governments and healthcare providers recognize the problem and predispose interventions to limit further loss of human life and any additional burden on a recovering healthcare systems and overstretched healthcare staff. [48, 49]. Stress response has evolved as a survival mechanism, enabling humans and other mammals to react quickly to life-threatening situations. Under chronic stress the body, by constantly feeling under attack, maintains a chronic activation of the stress response. Studies in different fields of biomedicine provide growing evidence that chronic stress, also at low level (*gutta cavat lapidem*),‘gets under the skin’ [50, 51]. Through the neuro-endocrine, cardiovascular and immune systems, it influences hormone release – e.g. cortisol and cholesterol levels, blood pressure and inflammation, eventually increasing the risk of mental and physical health conditions. [52–62]. Thus, chronic stress and insufficient recovery from stress are an increasing public health concern because of their long-term effects on health [63]. Similar to the 2008 financial crisis, the COVID-19 pandemic is a significant psychological stressor; fear of illness and uncertainty about the future precipitate anxiety and stress-related disorders. In addition, lockdown is expected to increase levels of loneliness, depression, harmful alcohol and drug use, post-traumatic syndrome and self-harm or suicidal behaviour [64–66]

As for the latter, The Italian Osservatorio dei suicidi reported 25 suicide during lockdown period, 16 only in the month of April, plus 36 suicide attempts. According to the Osservatorio in the same period, March, April 2019 the number of suicide was 14[67]. A similar increase was reported after the 2008 economic crisis, when the rate of suicide increased in European and American countries, particularly in males and in countries experiencing higher levels of job losses. According to Chung and coworkers there were an estimated 4884 (95% confidence interval 3907 to 5860) excess suicides in 2009 compared with the number expected based on previous trends (2000–07) [ 68, 69, 70, 71 ]. In addition to those dramatic detrimental behaviours, lockdown measures, by imposing social isolation, may increase loneliness, particularly amongst the elderly. Population-based studies have demonstrated that both objective social isolation and the perception of social isolation (loneliness) are correlated with a higher risk of mortality, and that both are clearly risk factors for cardiovascular disease (CVD) [72–87]. Within this picture, considering the direct and indirect burden of COVID-19 on mental health, a key component of well-being, the creation and dissemination of robust mental health screening and treatment programs for the general public and front-line healthcare workers is mandatory.

### What we measure affects what we do

While all over the world extraordinary effort are posed to keep the pandemics under some control hoping that it will soon disappear, questions arise: Have we learned the lesson that population health is the priority, no matter what? If the answer is yes, then in building strategies to preserve health in the future we need to use a “new culture of health”, a new paradigm. In our mind we tend to consider the concept of health only in relation to disease or illness, in other words we basically confine health, both at the individual and population level, within the frames of diseases and illness, physical and mental, thereby limiting health to being only a medical issue. However, when looking to the factors that determine health, we have found these include the conditions in which people are born, grow, live, work and age. Therefore, socioeconomic status, education, neighbourhood and physical environment, employment, and social support networks, as well as access to health care are strong “social determinants of health”. In addition, environmental factors like pollution, climate change, deforestation, to mention a few, are also “determinants of health”, and should be not considered as separate by social factors at this time, especially in reference to models of a circular economy which involve entire ecosystems that include society, governmental bodies and institutions, industry and investors.

### New investment models

Indeed, sustainability and corporate social responsibility generated a fervor of ideas, igniting profound organic and structural changes in different sectors; in particular, the increased sensitivity to the Environmental, Social and Governance (ESG) principles has led the financial system to question the needs of private and institutional investors, who could increasingly consider sustainable investments as a necessary prerogative in choosing their investments and in building their heritage.

In this regard, in recent years, banks’ awareness of the financial risks underlying climate change and potential stranded assets in the fossil fuel sector, and in those closely related, such as health, has increased, in parallel with the need to elaborate a new business model and a new strategy, promoting projects more in line with the decrease in global greenhouse gas emissions. An urge to consider the evaluation of any project investment, not only according to the economic-financial indicators but also to those that are declined in terms of “SRI” (Sustainable and Responsible Investment) or environmental, social and health governance criteria. This implies the requirement to take account of sustainability, as proposed by the UN agenda with its 17 Sustainable Development Goals introducing increasingly defined environmental, social, health and good governance criteria in the investment process.

These investment evaluation criteria are in line with EC Communication COM (2019) 640 - The European Green Deal, the new strategy for growth and to help European industry to guide the double transition to climate neutrality and digital leadership. Coherently, the EIB - European Investment Bank, from 2021 will cease to finance all projects in fossil sources, including gas, focusing on clean and renewable energy and will make available one trillion euros of sustainable investments in the sectors of the environment and climate action in the decade 2021–2030. Similarly, the major investment and pension funds are disinvesting from fossil fuels to invest in renewable energy, as fund managers believe that sustainable businesses and projects ensure higher longer-term returns. A sustainable finance strategy is more and more used in the context of investments by pension funds and large asset management companies, that have launched one of the most massive initiatives ever taken to strengthen and accelerate the necessary decarbonisation in the framework of the Paris Climate Agreement, directing investments towards low carbon activities, in order to have completely carbon neutral portfolios by 2050. Within this framework, their strategy looks for investments selected on the basis of compliance with international norms and standards defined in the UN, OECD or UNHCR, where the investment portfolio is chosen according to environmental, social and governance criteria and a holistic approach. Furthermore, the integration of ESG criteria in investments aims to maximize the long-term return of the portfolio, by better controlling the risks. In addition to obtaining financial returns, impact investing differs with respect to ESG criteria, as investors also want to have a calculable positive impact on the environment or society. The criteria for an investment for impact investing are:

- intentionality, that is, an explicit “*ex ante*” declaration and in the proactive search for activities that aim to create social value;
-measurability, the social impacts that are intended to be generated, as well as being established *ex ante*, must be identified in order to be measurable. Social objectives must in fact be measured with the aim of being able to define ex ante the expected impacts and ex post to verify whether the expected impacts have been effectively and effectively achieved;
-additionality, investments with a social impact take place in undercapitalized areas, or in those activities that would otherwise be excluded from any other investiture.

### Health, whatever it takes: depends on what we mean for health

Too often health is equated with healthcare, and although healthcare is essential to health, it is a relatively weak health determinant. In the ranking of relative weight of the various classifications of determinants of health the first place belongs to social determinants of health, 50–60%, adding environmental factors (10–20%) to social determinants of health the percentage rise to 70–80%. From this perspective “health as health care” is only a small part of “health”. In a broader and more comprehensive way, health is *a mean* to enable social, economic and personal development, *a resource* for everyday life. [88] Taken all together the determinants of health draw a painting whose title could be: Life. Our life, the quality of our life, how is our everyday life. Each of us hope to achieve a good life, to reach his/her well-being. Well-being is a cocktail of good and bad, of needs, of gender inequalities, of illness, of effort, of failure, of success, of inequalities, of expectations, of dreams, of future, of development, of poverty, of richness, of friendship, of social cohesion, of social polarization, of smiles, of tears, of joy, of grief. And each of us has his own share [89]-If we say health, but we intend well-being, it has to do with the whole societies not merely with the health systems. As mentioned previously, inequalities were on the rise since the 2008 financial crisis and the COVID-19 pandemic along with its social and economic consequence risks to dramatically worsen inequalities, which as Sir Michael Marmot [90] stated are “the cause of the cause of diseases”. An unequal social context harms health directly, also driving individuals into detrimental coping mechanisms and behaviours. Moreover, inequality harms health indirectly eroding societal trust and destabilizing communities, endangering social cohesion. The fact is that no one can be healthy in an unhealthy society. Whether a society is “healthy” or not cannot be analyzed and described only by macroeconomic statistics, such as GDP, in particular today the COVID-19 pandemic has unveiled the frailty of our economic systems. [91–92]. A “healthy” society is one which pursues societal improvements in the well-being of people and households. Successful reconstruction strategies must be built around the concept of wellbeing. Well-being measures material conditions (Income and Wealth, Housing, Work and Job), quality of life (Health, Knowledge and Skills, Environmental Quality, Safety) [93, −97] social support, relationships, sense of meaning, self-esteem, trust) and sustainability. Wellbeing distribution tells us about inequalities between population groups, between those at the top and those at the bottom of the achievement scale in each dimension and deprivation. Moreover, well-being directly links to resilience, the ability of people to adapt to change and to bounce back after illness and hardships. [98, 99, 100, 101]. In so doing wellbeing measures capturing and describing the “real life and needs” of people as these will be more useful particularly in informing successful strategies to address the COVID-19 pandemic crisis (or similar in the future), mitigate the post-crisis effects and probably prevent future crisis of this nature[102, −105]. What we measure affects what we do, and if we measure the wrong thing, we will do the wrong thing. If we focus only on material wellbeing – on, say, the production of goods, rather than on health, education, and the environment – we become distorted in the same way that these measures are distorted [37,38]. As stressed previously, this unprecedented situation has highlighted the need for carefully evaluating investment decisions at a macro level; decisions have to be taken after full consideration of all the alternatives, bearing in mind the cost of opportunity of each decision where the financial return to society may not be realised in pure monetary terms. A new system of indicators focusing on the health and well-being of human beings and not on profit can help ensure a real quality of life for all as opposed to simply generic and clumsy economic growth. A new model should take into account greater social cohesion and democratic participation, increase in volunteering, decrease in the percentage of citizens below the poverty line, real opportunities, better access to and quality of education, the number and quality of jobs created annually, acceptable productivity levels, greater availability of free time per capita, improved quality of the urban and extra-urban environment, the lowering of emissions, and the real life expectancy.

### Conclusions

At time we are writing, COVID-19 has been running the show world-wide, using human-beings as crazy fax machines, killing people, packing hospitals and ICU (where they are available), emptying cities, jeopardizing the economy and unveiling our frailties. As when COVID-19 from a bat or another wild animal to a human being and started to spread like wildfire, we were left astonished, and did not know how to face the unthinkable. Now we are not certain how to face the future. Unfortunately, time is not on our side: in the midst of the interpandemic stage we are entering, we have to rush to find solutions, and build our preparedness. The COVID-19 pandemic has nothing to do with this, it is just a litmus test, and for someone an alibi. We are totally responsible as we were perfectly aware that we were running towards a major disaster. We have been hesitating when defending democracy, which although imperfect is the best form of government we have, and overlooking the unrestrainable rise of poverty and inequalities in the Western civilized and developing countries. This has been translating to us neglecting the risk of social polarization, riding fear and ignorance, denying the systematic devastation of environment, turning our head when human rights were trampled on. Lately many words lost their meanings, such as “freedom”, that is primarily “responsibility”, respect, and recognizing everybody’s right and dignity: knowledge, is the grammar of development. Epidemics and pandemics have often changed the history of humanity for their demographic, economic and social effects they bring. We do not know whether the COVID-19 pandemic will change history or not, but one thing is certain, the post pandemic era will need an extraordinary socio-economic “reconstruction”.

We are observing the surge of American anger, generated by social unease and prolonged lockdown. Could it infect Europe, especially the peripheries of the metropolis? Lately an alarm bell rings on many sides about the risk that nationalism would be heralded as the perfect solution to inequalities and to the difficulties of so many people. This would be the worst possible answer to real problems that cannot longer be postponed. We have learned, and some still remember, that nationalisms have never been a solution, while they have always been nothing else than a devastating failure. As COVID-19 showed, we are all on the same boat, so the only reasonable answer should be a “reconstruction” strategy build on the bedrock of social cohesion and solidarity.

Any “reconstruction” strategies lacking a long term vision of a sustainable social and environmental development supported by a social and environmental sustainable economy, blindly focusing only on the production of goods, rather than on health, education, and the environment, will be the harbinger of new disasters and at that point there may be no hope left. In 1991 Howe and Russ in their book Generations, prophesied a global crisis for 2020, that the ruling classes would not be able to handle[106]. Perhaps to address the post COVID-19 crisis, we will need a new class of women and men with a long-term vision, able to use a totally new toolbox and gifted by cultural intelligence.

“Epidemics always test the limits of our societies and political imaginations, but history holds some unmistakable lessons: Societies further their own destruction whenever they fail to provide anyone with their physiological needs health care, nutrition, housing. Epidemics continue to remind us of our shared humanity because they show us how our individual survival is bound up in one another’s well-being” [107].
